# Aqueous solution and solid-state behaviour of l-homophenylalanine: experiment, modelling, and DFT calculations

**DOI:** 10.1039/d4ra01897d

**Published:** 2024-04-02

**Authors:** Vico Tenberg, Masoud Sadeghi, Axel Schultheis, Meenakshi Joshi, Matthias Stein, Heike Lorenz

**Affiliations:** a Physical and Chemical Foundations of Process Engineering Group, Max Planck Institute for Dynamics of Complex Technical Systems Magdeburg Germany sadeghi@mpi-magdeburg.mpg.de lorenz@mpi-magdeburg.mpg.de +49 391 6110 321; b Molecular Simulations and Design Group, Max Planck Institute for Dynamics of Complex Technical Systems Magdeburg Germany

## Abstract

In the present study, the solid-state and aqueous solubility behaviour of l-homophenylalanine (l-Hpa) is explored. Different characterization techniques such as TG, DSC, temperature-resolved PXRD, and hot-stage microscopy were used to investigate basic thermal solid-state characteristics. Solubilities of l-Hpa in water were determined as a function of temperature and pH. Moreover, a thermodynamic model based on perturbation theory (PC-SAFT) is applied to represent the data. In addition, aqueous density data of l-Hpa were measured in a broader temperature range. To model the solubility data as a function of pH, p*K*_a_ values are needed, which were accessed by employing density functional theory (DFT) calculations. The solid-state investigation did not show a simple melting process of l-Hpa, but a complete decomposition of the prevalent initial solid phase at elevated temperatures approximately above 520 K. This system exhibited extraordinarily low solubilities for an amino acid at all investigated temperatures. While the solubility does not differ from its isoelectric-point value over a wide pH range, it dramatically increases as the pH falls below 2.5 and rises above 9.5. The PC-SAFT model was able to calculate the solubilities as a function of pH and predict the density values.

## Abstract 2

In the present study, the solid-state and aqueous solubility behaviour of l-homophenylalanine (l-Hpa) is explored.

## Graphical abstract

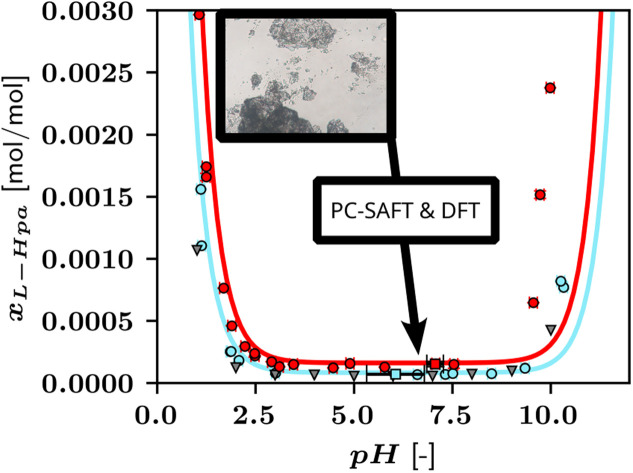

## 1. Introduction

Crystallization is widely used in industry to purify components, usually from liquid (solvent or melt) phases. For the design of efficient crystallization processes, fundamental knowledge about relevant solid–liquid equilibria (SLE) needs to be available. Especially in pharmaceutical applications, the product must be of high purity, due to possible health problems caused by impurities. However, solubility and solid-state form of the target component (*e.g.* presence of a solvate or polymorph) influence the potency as well.^1^ Therefore, SLEs are relevant not only for the crystallization itself but also for the complete production process.

Generally, either isothermal or polythermal measurements are used to determine the solubility of a substance.^2^ Different methods *e.g.* based on gravimetry,^3–6^ spectroscopy,^6,7^ titration,^8^ laser techniques,^9^ Differential Scanning Calorimetry (DSC),^10,11^ refractive index,^12,13^ or HPLC^14,15^ can then be applied as analytical techniques. Some of these methods have been in use for decades indicating their reliability.^3,4,16^ Depending on the availability of a specific compound or its manageability in lab scale experiments, a varying number of solubility data points are determined. To acquire additional data sets, either by inter- or extrapolation, modelling can be employed without the need for additional experiments, which, on the other hand, also can save elaborate and time-consuming lab work.

For more than two decades, we have been applying different solubility-measurement methods to investigate the solubility of different substances.^10,17,18^ This work focusses on the aqueous solution and solid-state behaviour of the rarely-investigated unnatural amino acid l-homophenylalanine (l-Hpa). It is employed as a precursor for production of various pharmaceutical drugs, *e.g.* for managing hypertension or congestive heart failure.^19^l-Hpa can be produced by different chemical or biocatalytic synthesis routes.^14,19^ Biocatalytic approaches have several advantages, such as enantioselectivity, feasibility near ambient conditions or being generally environmentally friendly and have therefore been studied in this respect.^19–22^ After synthesis, l-Hpa needs to be separated from the reaction mixture or further purified for which crystallization is an attractive separation technique due to its cost efficiency and the usually high product purity achievable. Aqueous solubilities of amino acids are dependent on temperature^3,15,16,23,24^ and pH-value.^15,25,26^ In our literature research, we only found one source for the solubility of l-Hpa in water and its dependence on pH which is represented in a graphical manner.^14^ To the best of our knowledge, no Powder X-Ray Diffraction (PXRD) or thermoanalytical study was published and therefore no diffractogram or melting properties are available at the time of writing. Further, many amino acids decompose before melting, making the measurement of melting properties impossible *via* common DSC.^27^ Nevertheless, melting properties are of great importance for thermodynamic modelling. Following, no publication including fundamental thermodynamic modelling and DFT calculations has been found for this system at the time of writing.

In this work, detailed aqueous solubilities of l-Hpa were measured in dependence of pH at two temperatures, namely 298 K and 328 K, to verify and expand available literature data.^14^ HPLC and PXRD analyses were used for evaluating the results of isothermal (static) experiments regarding liquid and solid phase characterization (composition, identity). Further, concomitant PXRD, thermogravimetry (TG) and DSC measurements as well as hot stage microscopy were applied to investigate the thermal solid phase behaviour of l-Hpa. The obtained solubility data were employed for thermodynamic modelling using Perturbed-Chain Statistical Associating Fluid Theory (PC-SAFT) Equation of State (EoS) for the estimation of activity coefficients. Various publications are available in the literature,^27–29^ among others, as well as from our previous works,^18,30^ which utilize PC-SAFT to model amino acid solution behaviour. As at the time of writing, p*K*_a_ values were not available, quantum chemical calculations were used to calculate the p*K*_a_ values of l-Hpa from isodesmic reactions in order to simulate the influence of pH on the solubility.

This paper is structured as follows: in the first part, section 2, experimental procedures and analysis methods will be described. Afterwards, in section 3, the theoretical background regarding thermodynamics and the selected models will be explained. Following that, in section 4, the obtained results are presented and discussed. Finally, concluding remarks of the present study as well as an outlook are given in section 5.

## 2. Experimental section

In the following section the chemicals, analysis and experimental methods will be described.

### 2.1. Materials

The applied chemicals are listed and specified in Table 1. The substances were used as received from the supplier. For some cases l-Hpa was further purified by recrystallisation. Water was purified using a Milli-Q Advantage device and had a total organic carbon content of 4 ppb with an electric resistivity of 18.2 MΩcm at 298 K.

**Table 1:** Detailed information of the chemicals used in this work

Material	CAS	Source	Purity	Molar mass (g mol^−1^)
l-Hpa	943-73-7	ThermoFisher (Kandel)	98%	165.19
Sodium carbonate	497-19-8	Merck	≥99.9%	105.99
Perchloric acid	7601-90-3	Merck	70–72%	100.46
Methanol	67-56-1	VWR International	99.8	32.04
Ammonium acetate	631-61-8	Sigma-Aldrich	98%	77.08
Acetic acid	64-49-7	VWR International	≥99.7%	60.05

### 2.2. TG and DSC analysis and hot stage microscopy

Thermogravimetric analysis in combination with differential scanning calorimetry allow to evaluate mass and enthalpy changes of a sample in parallel. TG was performed with a Sensys Evo twin type instrument manufactured by Setaram Instrumentation, France. Grinded samples of about 4 to 7 mg l-Hpa were weighed into open 100 μL aluminium crucibles and analysed with a linear temperature program under a helium (5.0) atmosphere. For the measurement, the following heating program was employed: starting at 298 K heating to 673 K with 2 K min^−1^ and subsequent cooling to 298 K with 5 K min^−1^. For DSC, a Setaram DSC 131 instrument was used. Grinded samples of about 6–8 mg were weighed into closed aluminium crucibles and analysed with the same temperature program than for TG under helium atmosphere. Temperature calibration of the instrument is performed regularly using the melting temperatures of highly pure indium, tin, zinc and lead at different heating rates.

To support interpretation of TG and DSC results, Hot Stage Microscopy (HSM) was performed using a hot stage LTS420 from Linkam Scientific Instruments, UK, combined with an Axioskop 2 microscope and an Axiocam 305 colour camera from Zeiss, Germany. A small sample was brought onto an object slide, covered by another glass slide and inserted into the hot stage. Then the sample was continuously heated to 607 K with a heating rate of 2 K min^−1^.

### 2.3. PXRD analysis at ambient and temperature-resolved conditions

Solids were analysed by PXRD with an X'Pert Pro diffractometer (PANalytical GmbH, Germany) with CuKα radiation in the 2*Θ* range from 4 to 30° with a step size of 0.017° and a counting time of 45 s per step. Solids from solubility experiments were analysed at ambient conditions. For temperature-resolved PXRD studies, pure l-Hpa was heated stepwise from 303 K to 523 K with a heating rate of 5 K min^−1^. At certain temperatures the sample was kept isothermal for a period of 3 min to reach thermal equilibrium, before the PXRD pattern is recorded with a measurement time of *ca.* 10 min. Afterwards, the sample was cooled down to 303 K at 5 K min^−1^. Solid samples were dried before analysis either in a fume hood or in a vacuum oven at 313 K at 200 mbar.

### 2.4. HPLC analysis

HPLC analysis was performed *via* a Dionex UltiMate3000 system manufactured by Thermo Scientific, Waltham. Two different HPLC methods were employed to quantify the l-Hpa concentration. The first method relies on a 150 mm long Crownpak (CR+) column (Daicel Corporation, Osaka) with a diameter of 4 mm and a particle size of 5 μm. Measurement took place at 298 K with an eluent flow rate of 0.8 mL min^−1^ and an injection volume of 1 μL. Detection was achieved by light absorbance at 200 nm. Aqueous solution of perchloric acid with a pH of 2 was used as eluent. This method was developed in our lab but a similar one was employed in the literature.^14^ The second method is based on another work,^31^ where a Chirobiotic T column (Astec) with a length of 250 mm, a diameter of 4.6 mm and a particle size of 5 μm was utilized. 0.5 mL per L acetic acid was added to 0.01 mol per L ammonium acetate solution, which was then mixed with methanol in the volumetric ratio of 30/70 to produce the eluent. Measurements were done with an eluent flow rate of 0.5 mL min^−1^ by adding 10 μL of sample and detection of light absorbance at 210 nm. Calibration was obtained by dissolving known amounts of l-Hpa in eluent and correlating the measured light absorbance to the concentration by linear regression.

Liquid samples were filtered (0.45 μm) and diluted with eluent; solid samples were dissolved completely in eluent. Alongside each measurement run, a sample with known concentration was analysed to verify the calibration.

### 2.5. pH determination

pH measurements were performed with an MP225 pH meter with an InLab Micro Pro probe manufactured by Mettler Toledo, USA. The system is calibrated in regular intervals and each day the accuracy was checked by measuring pH standards at room temperature. At 298 K the pH values were stable and used for the evaluation, while for 328 K either the stable value or the value after 60 s was accepted to reduce the influence of cooling (and potential nucleation).

### 2.6. Liquid density determination

For the determination of liquid densities, an equilibrated solution was produced by completely dissolving a known amount of l-Hpa in water at temperatures higher than the respective saturation temperatures. After a stirring period of at least 12 h to ensure complete dissolution, the sample was added into the density meter DM40 manufactured by Mettler Toledo, USA. During injection of the sample, it was ensured that no bubbles were present in the measurement tube prior to noting the final value. After achieving a temperature at which the sample is slightly undersaturated, the density was measured in 5 K steps until 353 K had been reached. After the last analysis, the sample was withdrawn and the device was flushed with water followed by ethanol and dried. Measurements have been done in triplicate in the beginning, but nearly no measurable scattering was observed. Therefore, most data points were measured once.

### 2.7. Solubility measurements

The procedures applied for determining the aqueous solubility of l-Hpa in dependence of temperature and pH were as follows:

Equilibrium experiments at isothermal conditions were performed in tempered double jacketed glass reactors at different temperatures. First, l-Hpa, was mixed with water and dissolved completely by heating the suspension. The samples of about 10 mL were then brought to the target temperature at which they were allowed to equilibrate for at least 48 h while being stirred continuously. Temperature control in the double jacketed glass reactors was conducted by thermocouples within an accuracy of ±0.1 K. After the equilibration period, the stirrer was switched off and the solid was left to settle. Then liquid was sampled and filtered (0.45 μm) two times by syringe filters: first, while sucking it into the syringe and second, after a fresh filter was applied when injecting into the eluent for HPLC measurement. For selected samples at both saturation temperatures (298 K and 328 K), a solid–liquid separation was conducted by vacuum filtration and the obtained solids were analysed by HPLC and PXRD.

Most experiments for determination of pH-dependent solubilities were performed in the Crystalline multi reactor system, manufactured by Technobis Crystallization Systems, The Netherlands. It contains eight glass reactors, which can be independently heated, stirred and observed with optical cameras and turbidity probes as well. Aqueous suspensions with l-Hpa content of 0.03 to 3 wt% were prepared at 328 K. After at least 10 min of stirring, a syringe pump added buffer (perchloric acid (7.66 or 0.08 wt%) or sodium carbonate (1.05 or 0.11 wt%) solution) continuously with a flowrate of 0.05 to 0.1 mL min^−1^. Due to the pH change, the solubility increases and the solid content in the reactors decreases. Shortly before complete dissolution was detected visually, the buffer addition was stopped. Then, the suspension was brought to target temperature and left stirring for approximately 20 h. On the next day, stirring was stopped, liquid samples were withdrawn and the pH was recorded. To determine the pI, the same procedure was performed without buffer addition.

To verify that equilibrium had been reached, experiments with longer equilibration times were performed. Therefore, l-Hpa and water were mixed and the buffer was added manually based on the results from previous experiments. After stirring at 298 K or 328 K for at least 21 d, liquid samples were taken *via* a syringe filter for HPLC analysis, and residual solid and liquid phases were separated by vacuum filtration to obtain solid phases for HPLC and PXRD analysis.

## 3. Theoretical section

Between distinct phases, which are at thermodynamic equilibrium, the chemical potential of any constituent i is the same in the phases present. In crystallization related systems, typically, a liquid phase is in equilibrium with a solid phase, which results in

1 *μ*^L^_i_ = *μ*^S^_i_

where *μ*_i_ is the chemical potential of compound i in the liquid L and solid S phase, respectively. The chemical potential can be expressed in terms of molar fraction *x*_i_, activity coefficient *γ*_i_, and the standard state fugacity *f*_i_^0^.^32^ Replacing the chemical potential in eqn (1) in such way and assuming a pure solid phase, results in2
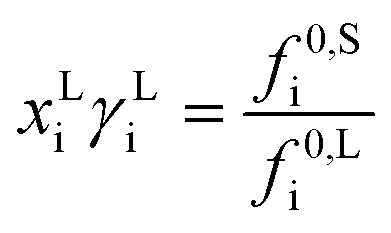


The standard state fugacities *f*^0,S^_i_ and *f*^0,L^_i_ are related to pure solid and pure subcooled liquid of compound i, respectively. Their ratio is usually determined as a function of melting temperature *T*_m,i_, molar melting enthalpy Δ*H*_m,i_, and the difference of the molar heat capacities of the pure solid and liquid phase Δ*C*_p,m,i_.3
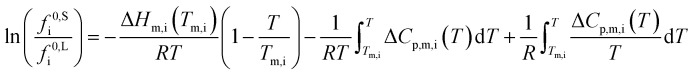
where *T* is the temperature and *R* = 8.314 J mol^−1^ K^−1^ is the universal gas constant.

To calculate the solubility of compound i in a given solvent (*x*^L^_i_), its activity coefficient must be determined. In this work, the activity coefficient is predicted using the PC-SAFT EoS, which is explained in greater detail in section 3.1.

The influence of pH on the solubility is modelled as a factor to the solubility of the solute in a single solvent at the isoelectric point pI.^33^4
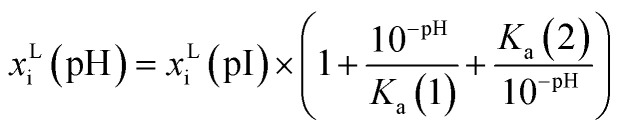
where *K*_a_ is the acid ionization constant and can be calculated from p*K*_a_.

5 *K*_a_(*j*) = 10^−p*K*_a_(*j*)^

In this work, p*K*_a_ values were determined using Density Functional Theory (DFT) with an implicit solvent model, which is further detailed in section 3.2.

### 3.1. Perturbed-chain statistical associating fluid theory model

The PC-SAFT model treats molecules as chains of connected hard spheres of specific length and sphere sizes depending on the molecule.^34^ These chains interact in various ways with other chains, either with identical chains representing the same molecule or with different molecules and their respective chains. Such chains are characterized by various parameters such as number of chain segments *m*, temperature-independent segment diameter *σ*, and a parameter for the dispersion energy between two identical chains *u*/*k*. Here, *k* = 1.381 × 10^−23^ J K^−1^ is the Boltzmann constant.

For associating compounds, two additional parameters are required.^35^ These are the parameters representing association energy *ε*^A_i_B_i_^/*k* and association volume *κ*^A_i_B_i_^ between association sites A and B of compound i. The interaction parameters of chains representing different molecules can be described with the following mixing rules:^34,35^6
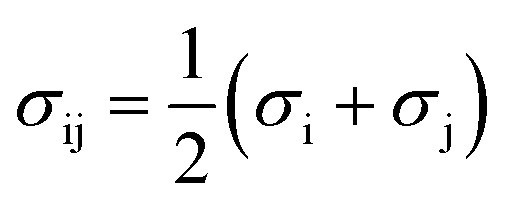
7
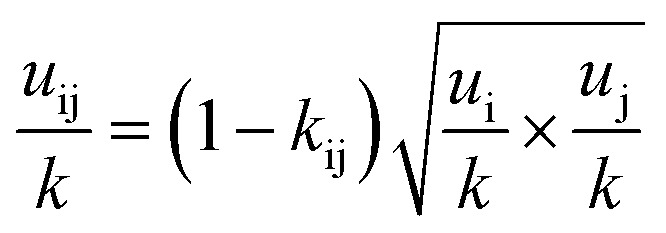
8
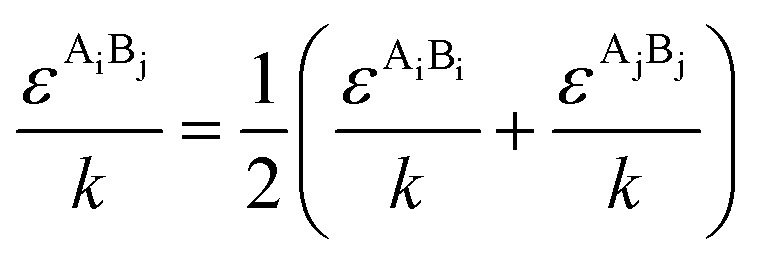
9
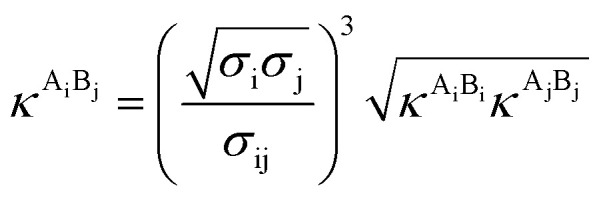
where *k*_ij_, in eqn (7), is the dispersion energy correction parameter. In this work, this potentially temperature dependent parameter is modelled as follows:

10 *k*_ij_ = *k*_ij,*T*_0__ + *k*_ij,*T*_(*T* − *T*_0_)

Its parameters *k*_ij,*T*0_ and *k*_ij,*T*_ are fitted to experimental data sets for *T*_0_ = 298.15 K.

Using these pure component parameters as well as their mixture values, the compressibility factor *Z* (eqn (11)) is calculated. It is composed of the hard chain *Z*_hc_,^34^ dispersion *Z*_disp_^34^ and association contributions *Z*_assoc_.^36^ The underlying equations for these contributions can be found in their above-mentioned citations.

11 *Z* = 1 + *Z*_hc_ + *Z*_disp_ + *Z*_assoc_

From the compressibility factor *Z*, it is possible to calculate the fugacity coefficient *φ*_i_, which is dependent on the temperature *T*, pressure *p* as well as the molar fractions *x* of the components in the system.^36^12
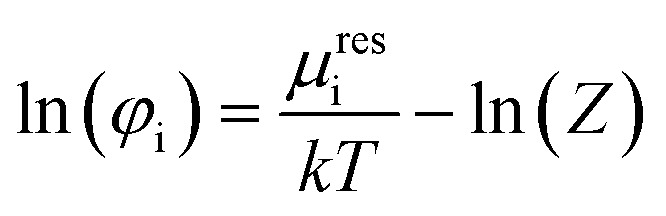


The calculation of the residual chemical potential *μ*^res^_i_ is given in the literature.^34^ Relating the fugacity coefficient in a mixture to its value for a pure compound i, yields the activity coefficient *γ*_i_.13
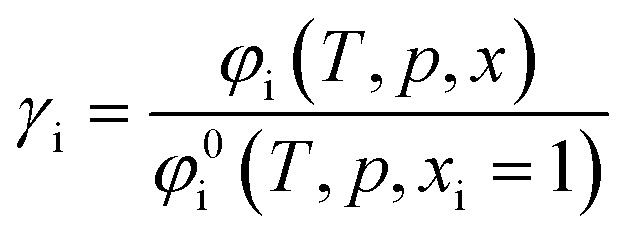


### 3.2. Determination of p*K*_a_

DFT calculations were performed to determine the p*K*_a_ of l-Hpa using Turbomole v. 7.5.1.^37^ The amino acid structures were optimized in the gas phase using the BP86 and PBE0 functionals, def2-TZVP basis sets and the RI approximation.^38–41^ Frequency calculations were carried out at the same level of theory to obtain thermodynamic corrections at 298.15 K. Solvent effects were incorporated using the COSMO solvation model^42,43^ and Grimme's D3 dispersion correction^44^ was applied in all calculations. Structures were fully re-optimized in the COSMO environment.

**Scheme 1:**
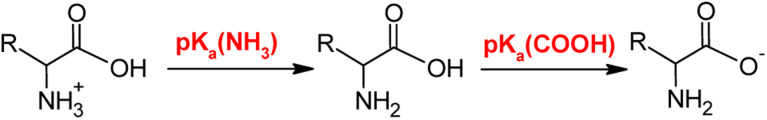
Sequential ionization reactions considered for the p*K*_a_ calculation of amino acids.

The p*K*_a_ of phenylalanine was calculated to validate the accuracy of the chosen method. Scheme 1 was used to calculate the p*K*_a_ values of the COOH [p*K*_a_(1)] and NH_3_ [p*K*_a_(2)] groups in these amino acids.

**Scheme 2:**
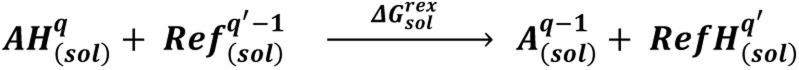
Isodesmic reaction of proton exchange between amino acid of interest (AH) and a reference acid molecule (histidine, represented as RefH). The charges on acids and their conjugate base are represented by *q*/*q*′ and *q* − 1/*q*′ − 1.

An isodesmic reaction approach was employed for the purpose of calculating p*K*_a_ values as illustrated in Scheme 2.^45^ The calculations were conducted using different reference acids; with histidine as a reference, best p*K*_a_ values for phenylalanine could be obtained. The Gibbs energy of the proton exchange reaction (Δ*G*^rex^_sol_) was subsequently calculated using Scheme 2.

Finally, the p*K*_a_ values of l-Hpa and phenylalanine were calculated using eqn (14). For the reference acid (histidine), experimentally reported p*K*_a_ values: p*K*_a_(1) of 1.64 ± 0.45 for the COOH group and p*K*_a_(2) of 9.14 ± 0.08 for the NH_3_ group^46^ were used.14
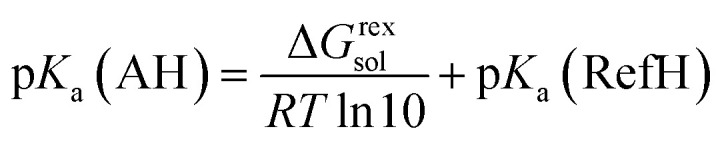


## 4. Results and discussions

### 4.1. Thermal solid phase behaviour of l-Hpa

**Fig. 1:**
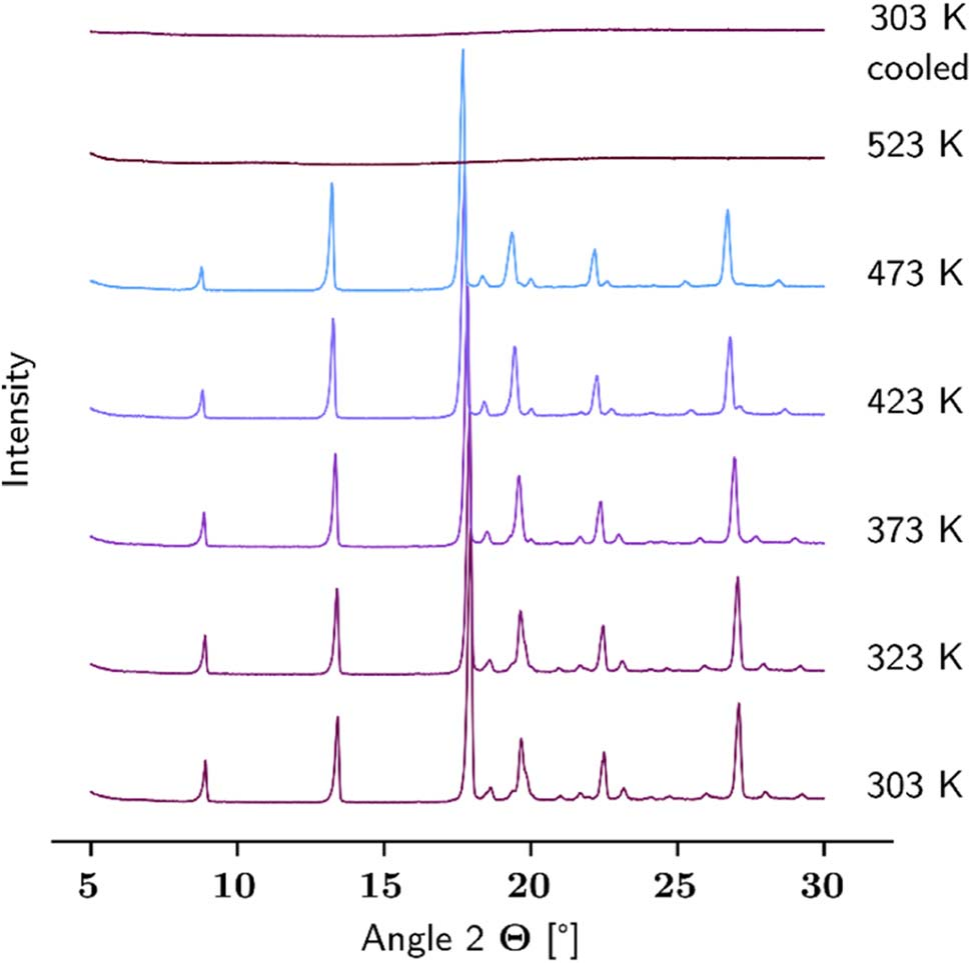
Temperature-resolved PXRD patterns of l-Hpa (as received) between 303 K and 523 K, and after re-cooling to 303 K (from bottom to top).

At the time of writing and to the best of our knowledge, no information about the thermal behaviour of solid l-Hpa is available in literature. Therefore, this chapter aims to deliver first insights into the thermal and solid phase characteristics of this compound. Initially, a temperature-resolved PXRD analysis was performed. The resulting diffractograms are given in Fig. 1, starting with the powder pattern of the l-Hpa sample at 303 K, followed by those at elevated temperatures up to 523 K and ending with the final one after cooling down to 303 K (from bottom to top). The supplied l-Hpa at 303 K shows sharp diffraction peaks indicating crystalline behaviour. Characteristic peaks can be observed at 8.9, 13.4, 17.9, 19.7, 22.5, and 27.1° alongside other peaks of lesser intensity. During heating up to a temperature of 473 K, no obvious significant change in the pattern is obtained, verifying the basic stability of the prevalent crystalline phase in the studied temperature range. Nevertheless, a few smaller peaks gradually disappear at 19.4, 21.0, 21.7, and 28.0° and at least one new evolves at 20.0° mainly between 373 K and 473 K.

At 523 K no crystalline substance is left, and upon cooling the sample down to 303 K, no recrystallisation occurred as the diffractogram remains basically unchanged.

**Fig. 2:**
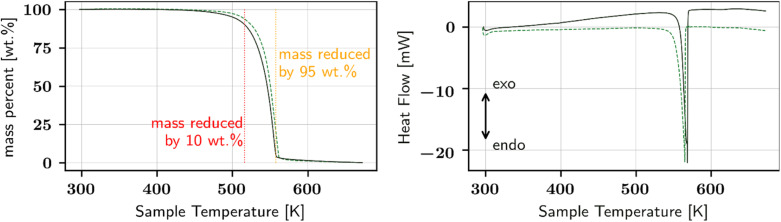
Left: TG curves of l-Hpa, given as the relative mass change referred to the initial sample mass. Solid line: recrystallized l-Hpa, dashed line: as received. Right: DSC curves of l-Hpa as received: dashed line, and recrystallized: solid line (6 mg and 7.4 mg, respectively). Red vertical dotted line: temperature where 10 wt% mass has been lost in TG-measurement of the recrystallized l-Hpa, orange vertical dotted line: temperature where 95 wt% have been lost.

To further interpret the revealed phase behaviour, TG and DSC measurements were performed. Here, the sample was heated from 298 K to 673 K with a heating rate of 2 K min^−1^, followed by cooling down with 5 K min^−1^. Fig. 2 illustrates the resulting TG and DSC curves represented as the relative mass loss occurring on the sample (left) detected by the Sensys Evo, and the heat flow (right) measured in DSC 131. Only the heating run is illustrated. The dashed lines refer to l-Hpa as received, while solid lines represent l-Hpa recrystallized from solution. In general both samples show comparable thermal behaviour. A slight loss of mass starts at about 450 K reaching *ca.* 10 wt% at about 520 K, followed by a strong and almost complete mass loss of 95 wt% up to about 560 K, leaving behind a small amount of black residue in the crucibles after the TG measurement. The DSC curves exhibit a strong and narrow endothermal peak between about 550 K and 570 K (with peak onset and peak maximum temperatures at 550/556 K and 565/568 K for the initial and recrystallized sample, respectively), which is connected with a huge heat consumption far from the magnitude of a melting effect (617 J g^−1^ for the recrystallised sample). Thus, a simple melting behaviour can be excluded for l-Hpa under the conditions used; instead decomposition and pyrolysis processes occur.

**Fig. 3:**
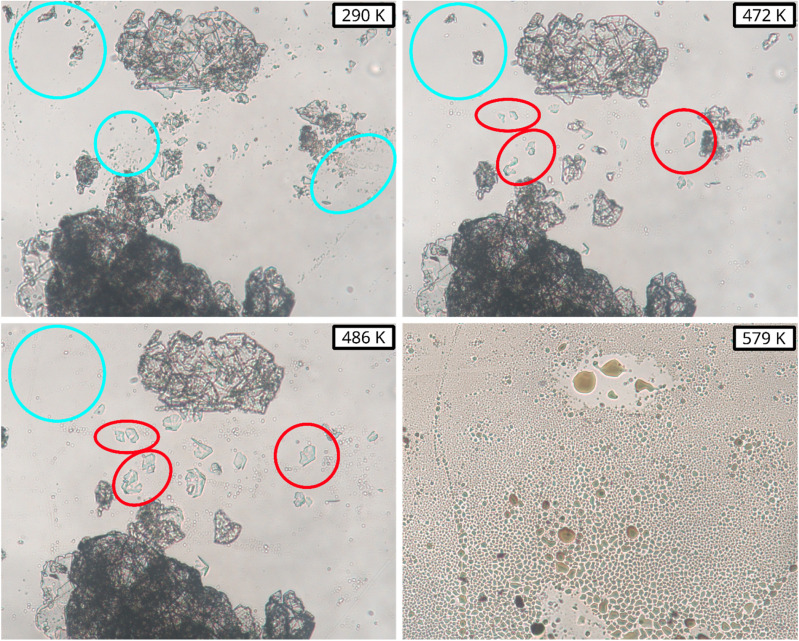
Selected pictures taken during hot stage microscopy of recrystallized l-Hpa. The respective temperatures are included in the figure. Disappearing crystals are highlighted with blue ellipses, and emerging or growing crystals with red ellipses.

To further investigate the thermal behaviour, HSM was utilized. Fig. 3 illustrates pictures of the sample material at different temperatures of an exemplary heating run. With heating from 289 K to 486 K, a slight decrease in small present particles is observed and, contrariwise, new particles appear and grow (see blue and red circles, respectively, in Fig. 3). Further heating causes the appearance of small gas bubbles and the sample decomposes leaving behind a yellow-brown sticky liquid residue already at 520 K (not shown; compare picture at 578 K). The latter is in agreement with the findings from the TG and DSC study, where the samples undergo a strong decomposition-based mass loss in the mentioned temperature range. It also supports the temperature-resolved PXRD measurement, showing full loss of crystallinity at 523 K. How far the disappearing and newly appearing crystals correlate with the few small gradually disappearing and the novel emerging PXRD peaks cannot be clearly stated at present time, and is out-of-scope of this paper. Ostwald ripening and formation of a new solid phase are potential explanations among others. Also, the impurity spectrum of the l-Hpa material as received has been indicated to play a role.

Concluding, the employed analysis methods (PXRD, TG, DSC, and HSM) show coherent results to each other, although some details need to be investigated deeper in future work.

### 4.2. Solubility behaviour of l-Hpa in aqueous solution

In this work, l-Hpa solubilities and densities in water were determined to extend existing literature data.^14^ For this, solubilities and densities of various undersaturated solutions were measured at various temperatures following the experimental procedures outlined in sections 2.6 and 2.7.

To model the determined data sets, several pure and interaction parameters are required. For l-Hpa, these parameters were fitted to our experimental data sets using the PC-SAFT model. As detailed in section 4.1, melting properties for l-Hpa are not available, as it is the case for many amino acids. Therefore, in this work, they were fitted to experimental data sets as well and should be revised if accurate melting properties become available *e.g. via* elaborate FSC measurements.^27^ The molar heat capacity difference between pure liquid and solid l-Hpa was neglected in this work, thus Δ*C*_p,m_ = 0 holds. In the model, *N*_assoc_ = 2 (one donor and one acceptor site) was used as the number of association sites for both molecules. All parameters were fitted to solubility data using the following objective function (OF):15
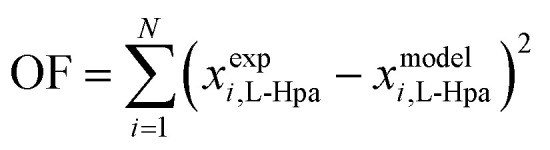


For water, pure component parameters for PC-SAFT are available in literature^47^ and are listed alongside the fitted parameters for l-Hpa in Table 2.

**Table 2:** Component specific parameters for PC-SAFT used in this work

Component	*m*	*σ* (A)	*u*/*k* (K)	*ε* ^A_i_B_i_^/*k* (K)	*κ* ^A_i_B_i_^	*k*_ij,298.15 K_(H_2_O)	*k*_ij,*T*_(H_2_O)	Ref.
l-Hpa	5.0448	3.0444	331.999	3860.9999	0.0304	0.0018	4.7 × 10^−4^	This work
Water	1.2046	a	353.9449	2425.6714	0.0451	0.0000	0.0000	47

a *σ* = 2.7927 + 10.11 exp(−0.01775*T*) − 1.417 exp(−0.01146*T*).

The parameter fitting resulted in a (virtual) melting temperature of *T*_m,l-Hpa_ = 584.15 K and a (virtual) molar melting enthalpy of Δ*H*_m,l-Hpa_ = 31 994.99 J mol^−1^.

**Fig. 4:**
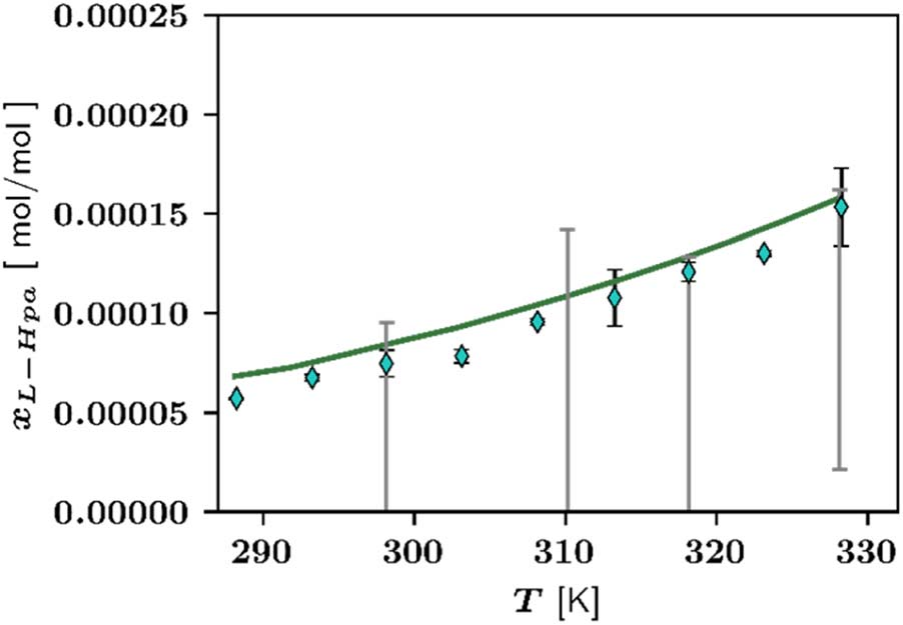
Solubility of l-Hpa in water at various temperatures from 288 K to 328 K. Experimental data points from this work ◊ and grey bars from literature.^14^ For our own data points, error bars are also included. Green line is calculated using the PC-SAFT model. Note that the literature data were measured at pH 7.5.

Fig. 4 displays temperature-dependent l-Hpa solubilities in pure water resulting from experimental and theoretical investigations.

As observable from Fig. 4, the overall solubility of l-Hpa in water increases with temperature but is extremely low when compared to other amino acids.^27^ Our measurements resulted in solubilities between 0.0075 mol% at 298 K and 0.0153 mol% at 328 K. In absolute numbers, these values are by trend slightly higher than data reported in a previous work by Cho *et al.*^14^ Due to the very low overall solubility and therefore challenging experimental work, a larger relative error is to be expected in solubility determination, which might be a possible explanation for these deviations. Further, the literature data were determined at a different pH and, in addition, extracted from a figure and might thus deviate from the actual measured values. Since we repeated our measurements several times in different time periods and obtained very similar results, we take our measurements as realistic values.

Additionally, Fig. 4 shows the solubility resulting from the modelling with PC-SAFT. Overall, the model and experimental data sets agree well with each other.

One could argue, that fitting the PC-SAFT related parameters and melting properties simultaneously might affect the integrity of the values. Alternatively, PC-SAFT parameters could be fitted to data sets – independent of melting properties – such as solution densities.

**Fig. 5:**
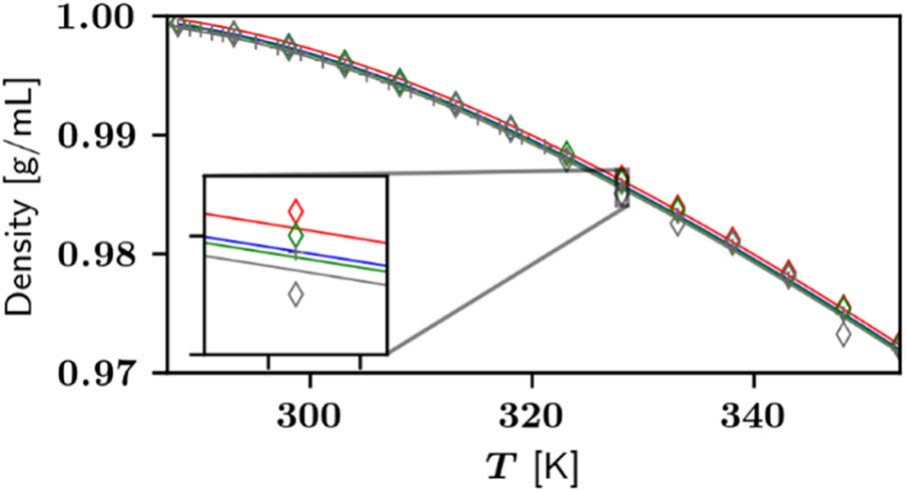
Liquid phase densities of l-Hpa solutions with different concentrations of 18.0 × 10^−5^ in red, 8.2 × 10^−5^ in blue, and 5.7 × 10^−5^ in green in water at various temperatures from 288 K to 353 K. Pure water data are shown in grey. Experimental data points from this work ◊. Lines are predicted by PC-SAFT. Density of water from literature^48^ are shown as +.

To validate PC-SAFT parameters – which were fitted to solubility data – solution density modelling were used. Fig. 5 illustrates temperature-dependent densities for an undersaturated solution containing *x*_l-Hpa_ = 5.7 × 10^−5^ mol per mol l-Hpa in water.

Overall, the modelled data agrees well with the experimentally determined solution densities, although, the density data is slightly underpredicted by the model. Additionally, the model is able to predict density data at temperatures higher than 328 K, which was the highest temperature used in parameter fitting. Measurement and prediction for various undersaturated solutions verified that the model is able to predict the correct trend of increasing density with increasing solute concentration. Based on these results, we assume our fitted PC-SAFT parameters to be reasonable. However, it should be noted that since the l-Hpa fraction is very small compared to the water fraction, its influence on the solution density is diminished. This again, leads to the conclusion, that model parameters reported in this work should be re-evaluated once accurate melting properties are available.

### 4.3. p*K*_a_ values of l-Hpa

In Table 3 the calculated p*K*_a_ values of the COOH [p*K*_a_(1)] and NH_3_ [p*K*_a_(2)] of phenylalanine and l-Hpa are given together with experimental data reported for phenylalanine.^46^ The BP86 calculated p*K*_a_(1) value of phenylalanine exhibits an error of approximately 0.39 units relative to the corresponding experimental value. However, the calculated p*K*_a_(2) shows a larger error of approximately 0.99 unit. In particular, p*K*_a_(1) is slightly underestimated, while p*K*_a_(2) is overestimated. The deviation can be attributed to the chosen reference value of histidine. We can anticipate a similar error in the calculated p*K*_a_ of l-Hpa [p*K*_a_(1) = 2.39 and p*K*_a_(2) = 10.04].

**Table 3:** Calculated values of p*K*_a_ of phenylalanine and l-Hpa using BP86-D3 [PBE0-D3] functionals and def2-TZVP basis set. Reported experimental p*K*_a_ values of phenylalanine are also included^46^

Amino acids	p*K*_a_(1)	p*K*_a_(2)
l-Homophenylalanine (calc.)	2.39 [2.25]	10.04 [10.18]
Phenylalanine (calc.)	1.82 [1.78]	10.16 [10.05]
Phenylalanine (exp.^46^)	2.21 ± 0.15	9.17 ± 0.06

### 4.4. Influence of pH on aqueous solubilities of l-Hpa

**Fig. 6:**
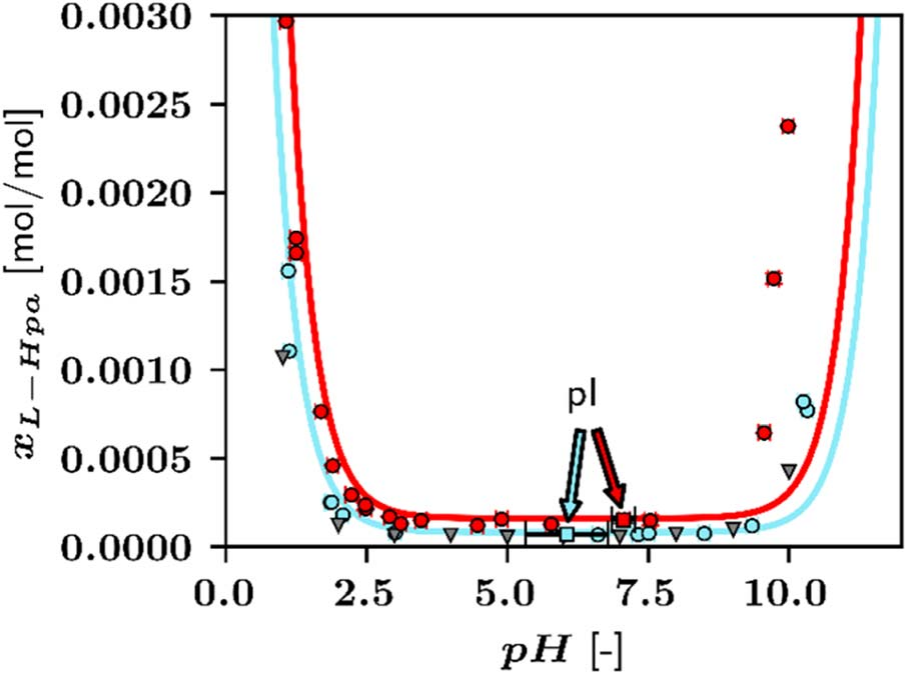
Solubility of l-Hpa in water at various pH-values between 298 K and 328 K. Experimental data points from this work ○, 298 K: blue, 328 K: red, data from ref. 14 ▼ at 310 K in grey. Lines are calculated applying the PC-SAFT model and eqn (4). pIs are indicated using arrows and the markers ■.

In addition to l-Hpa solubilities in pure water, solubility data sets in presence of basic and acidic buffers at 298 K and 328 K were determined during this work. For buffers, sodium carbonate and perchloric acid were used as basic and acidic buffers, respectively. The choice of buffers will affect the solubility of the solute slightly, however, due to the low concentrations of these compounds, this effect was not further investigated and neglected in the solubility modelling. In section 4.3, p*K*_a_-values for l-Hpa were investigated to be p*K*_a_(1) = 2.39 and p*K*_a_(2) = 10.04. Thus, the pI of l-Hpa was identified to be 6.40. Fig. 6 presents the determined pH-dependent solubility data of l-Hpa in water compared to literature results.

The increase of solubility at lower and higher pH-values, typical for amino acids as ampholytes,^5,12^ starts for l-Hpa at a particular low pH of about 2 and high pH of 9.5. Thus, l-Hpa exhibits an unusually broad section where the solubility is not affected by the pH. The highest solubility was observed at pH 1.07 at 328 K with a value of 0.278 mol%, which is by a factor of 18.2 higher than the solubility at the pI.

From the solubility minimum, the pI was determined to be 6.06 at 298 K, however, due to the mentioned broad base of the U-shape, individual data points exhibit significant scattering. In the literature,^14^ aqueous l-Hpa solubility was reported at 310 K, using sulfuric acid and sodium hydroxide as buffers. Even though slightly different conditions were investigated, a comparable broad base and pH-dependent solubility increase were observed. As represented by the solid line in Fig. 6, our modelling approach is able to predict the experimental data sets well for pH < 9.5. The solubility increase at higher pH-values is underpredicted to some extent by the model and leading to a lower pH-dependency than the actual data. At 328 K, the general behaviour is similar but the pI shifts to pH 7.07 with a solubility of 0.015 mol%. This is seen as a reasonable result, since the pH-dependence in the model is a function of just p*K*_a_. Alternatively, one could implement electrolyte contributions into PC-SAFT to incorporate the influence of specific buffer compounds.^14^ Additionally, p*K*_a_ was treated as temperature-independent in this work. Therefore, further accuracy could be achieved by taking into account the p*K*_a_ change with temperature using the protonation enthalpy.^33^

## 5. Conclusion

This work investigated the SLE in aqueous solutions and the thermal solid phase behaviour of l-Hpa. TG, DSC, temperature-resolved PXRD and hot stage microscopy experiments showed that l-Hpa does not exhibit simple melting behaviour but significant decomposition *via* pyrolysis processes above *ca.* 520 K. Experimental efforts showed that the aqueous solubility of l-Hpa is very low with 0.0075 mol% at 298 K, and increases to 0.015 mol% if the temperature rises to 328 K. Also, density values of undersaturated solutions only differ slightly from values of pure water due to the low solubility of l-Hpa. PC-SAFT is able to represent the solubility behaviour and predict the density values. The solubilities remain practically unchanged over a wide range of pH. However, at sufficiently low (1.5) or high (10.5) pH, solubilities increase almost twentyfold. DFT calculations showed a promising result for the calculation of the l-Hpa ionization constant. With this, pH-dependent solubilities were predicted well for pH-values below 9.5.

Further efforts need to be invested into more clarification of the thermal solid phase behaviour of l-Hpa, which helps to revise the model parameters such as (virtual) melting data. Additional experiments should be conducted to cover further conditions, especially for pH-dependent solubilities, as only two temperatures were investigated in this work.

Concluding, this work provides the fundamental SLE required to design a crystallization-based purification process for l-Hpa from aqueous solution. Based on the results, a pH shift crystallization appears to be a reasonable strategy that is favourable in terms of prospective yields and productivity.

## Author contributions

Vico Tenberg: methodology, modelling, validation, formal analysis, visualization, writing – original draft, writing – review & editing, supervision. Masoud Sadeghi: conceptualization, methodology, modelling, validation, formal analysis, investigation, visualization, writing – original draft, writing – review & editing, supervision. Axel Schultheis: methodology, validation, formal analysis, investigation, visualization, writing – original draft. Meenakshi Joshi: methodology, modelling, investigation, writing – original draft, writing – review & editing. Matthias Stein: writing – review & editing, resources. Heike Lorenz: conceptualization, supervision, writing – original draft, writing – review & editing, resources.

## Conflicts of interest

There are no conflicts to declare.

## Supplementary Material

## References

[cit1] Di L., Fish P. V., Mano T. (2012). Drug Discovery Today.

[cit2] Lorenz H. , in Crystallization: Basic Concepts and Industrial Applications, Wiley, 2013, pp. 35–74

[cit3] Wang J., Li J., Liu D., Wang Y., Zhang S., Liu Y., Zhao L., Xing X., Zhi M., Wang P., Hou X. (2023). J. Chem. Eng. Data.

[cit4] Sing N., Mondal P., Mondal M., Mahali K., Henaish A. M. A., Saha B., Ahmed J., Hussain A., Roy S. (2023). Chem. Phys. Lett..

[cit5] Saha A., Mahali K., Ganai S., Mukherjee P., Shrestha N. K., Henaish A. M. A., Ahmed J., Kundu S., Roy S. (2023). J. Mol. Liq..

[cit6] Aliyeva M., Brandão P., Gomes J. R. B., Coutinho J. A. P., Held C., Ferreira O., Pinho S. P. (2023). J. Chem. Thermodyn..

[cit7] Saikia J., Devi T. G., Karlo T. (2023). J. Mol. Struct..

[cit8] Roy S. (2022). Russ. J. Phys. Chem. A.

[cit9] Ma J., Li H., Jia J., Wang W., Liu W., Yao X., Li T., Ren B. (2023). J. Mol. Liq..

[cit10] Mohan R., Lorenz H., Myerson A. S. (2002). Ind. Eng. Chem. Res..

[cit11] Sapoundjiev D., Lorenz H., Seidel-Morgenstern A. (2005). Thermochim. Acta.

[cit12] Aliyeva M., Brandão P., Gomes J. R. B., Coutinho J. A. P., Ferreira O., Pinho S. P. (2022). J. Chem. Eng. Data.

[cit13] Naderi S., Ebrahimi N., Sadeghi R. (2023). J. Chem. Thermodyn..

[cit14] Cho B.-K., Seo J.-H., Kim J., Lee C.-S., Kim B.-G. (2006). Biotechnol. Bioprocess Eng..

[cit15] Liu L., Jia L., Yang W., Xiao Y., Dai J., Cui P., Zhou L., Yin Q. (2022). J. Solution Chem..

[cit16] Dunn M. S., Ross F. J., Read L. S. (1933). J. Biol. Chem..

[cit17] Lorenz H., Sapoundjiev D., Seidel-Morgenstern A. (2003). Eng. Life Sci..

[cit18] Sadeghi M., Tenberg V., Münzberg S., Lorenz H., Seidel-Morgenstern A. (2021). J. Mol. Liq..

[cit19] Ahmad A. L., Oh P. C., Shukor S. R. A. (2009). Biotechnol. Adv..

[cit20] Heuson E., Charmantray F., Petit J.-L., de Berardinis V., Gefflaut T. (2019). Adv. Synth. Catal..

[cit21] Wu T., Mu X., Xue Y., Xu Y., Nie Y. (2021). Biotechnol. Biofuels.

[cit22] Li H., Liao J. C. (2013). ACS Synth. Biol..

[cit23] Tahri Y., Gagnire E., Chabanon E., Bounahmidi T., Mangin D. (2016). J. Cryst. Growth.

[cit24] Mishelevich A., Apelblat A. (2008). J. Chem. Thermodyn..

[cit25] Voges M., Prikhodko I. V., Prill S., Hübner M., Sadowski G., Held C. (2016). J. Chem. Eng. Data.

[cit26] Lee C.-Y., Chen J.-T., Chang W.-T., Shiah I.-M. (2013). Fluid Phase Equilib..

[cit27] Do H., Chua Y., Kumar A., Pabsch D., Hallermann M., Zaitsau D., Schick C., Held C. (2020). RSC Adv..

[cit28] Grosse Daldrup J.-B., Held C., Ruether F., Schembecker G., Sadowski G. (2010). Ind. Eng. Chem. Res..

[cit29] Held C., Cameretti L. F., Sadowski G. (2011). Ind. Eng. Chem. Res..

[cit30] Tenberg V., Hokmabadi M., Seidel-Morgenstern A., Lorenz H., Sadeghi M. (2023). Ind. Eng. Chem. Res..

[cit31] Verseck S., Bommarius A., Kula M. R. (2001). Appl. Microbiol. Biotechnol..

[cit32] Prausnitz J. M. , Lichtenthaler R. N. and de Azevedo E. G., Molecular Thermodynamics of Fluid Phase Equilibria, Wiley-VCH, Weinheim, 1993

[cit33] Rüther F. and Sadowski G., in Industrial Scale Natural Products Extraction, Wiley-VCH, 2011, pp. 27–54, 10.1002/9783527635122.ch2

[cit34] Gross J., Sadowski G. (2001). Ind. Eng. Chem. Res..

[cit35] Gross J., Sadowski G. (2002). Ind. Eng. Chem. Res..

[cit36] Chapman W., Gubbins K., Jackson G., Radosz M. (1990). Ind. Eng. Chem. Res..

[cit37] TURBOMOLE V7.5.1 2021, a development of University of Karlsruhe and Forschungszentrum Karlsruhe GmbH, 1989-2007, TURBOMOLE GmbH, since 2007, https://www.turbomole.com, accessed 23-09-13

[cit38] Perdew J. P., Yue W. (1986). Phys. Rev. B: Condens. Matter Mater. Phys..

[cit39] Ernzerhof M., Scuseria G. E. (1999). J. Chem. Phys..

[cit40] Weigend F., Ahlrichs R. (2005). Phys. Chem. Chem. Phys..

[cit41] Eichkorn K., Weigend F., Treutler O., Ahlrichs R. (1997). Theor. Chem. Acc..

[cit42] Klamt A., Schüürmann G. (1993). J. Chem. Soc., Perkin Trans. 2.

[cit43] Schäfer A., Klamt A., Sattel D., Lohrenz J. C. W., Eckert F. (2000). Phys. Chem. Chem. Phys..

[cit44] Grimme S., Antony J., Ehrlich S., Krieg H. (2010). J. Chem. Phys..

[cit45] Casasnovas R., Ortega-Castro J., Frau J., Donoso J., Muñoz F. (2014). Int. J. Quantum Chem..

[cit46] Henchoz Y., Schappler J., Geiser L., Prat J., Carrupt P.-A., Veuthey J.-L. (2007). Anal. Bioanal. Chem..

[cit47] Veith H., Luebbert C., Sadowski G. (2020). Cryst. Growth Des..

[cit48] Kretzschmar H.-J. and Wagner W., in VDI-Wärmeatlas: Fachlicher Träger VDI-Gesellschaft Verfahrenstechnik und Chemieingenieurwesen, ed. P. Stephan, S. Kabelac, M. Kind, D. Mewes, K. Schaber and T. Wetzel, Springer Berlin Heidelberg, Berlin, Heidelberg, 2019, pp. 201–218, 10.1007/978-3-662-52989-8_12

